# Human Cytomegalovirus UL23 Attenuates Signal Transducer and Activator of Transcription 1 Phosphorylation and Type I Interferon Response

**DOI:** 10.3389/fmicb.2021.692515

**Published:** 2021-07-09

**Authors:** Linyuan Feng, Wanwei Li, Xingyuan Wu, Xiaotian Li, Xiaoping Yang, Yanhong Ran, Jianguo Wu, Hongjian Li

**Affiliations:** ^1^Department of Biotechnology, College of Life Science and Technology, Jinan University, Guangzhou, China; ^2^Guangdong Provincial Key Laboratory of Virology, Institute of Medical Microbiology, Jinan University, Guangzhou, China; ^3^Foshan Institute of Medical Microbiology, Foshan, China

**Keywords:** HCMV tegument protein, UL23, human cytomegalovirus, IFN-stimulated genes, immune escape, innate immune response, STAT1 phosphorylation, Type I interferon

## Abstract

Human cytomegalovirus (HCMV), the human beta-herpesvirus, can cause severe syndromes among both immunocompromised adult patients and newborns. Type I interferon (IFN-I) exerts an important effect to resist infections caused by viruses such as HCMV, while IFN evasion may serve as a key determining factor for viral dissemination and disease occurrence within hosts. In this study, UL23, a tegument protein of HCMV, was confirmed to be a key factor for negatively regulating the type I IFN immune response. A detailed analysis indicated that the viral UL23 protein increases the IFN-I antiviral resistance during HCMV infections. Furthermore, UL23 was shown to significantly reduce the levels of IFN-stimulated genes (ISGs) and promoter activity of IFN-I-stimulated response element. Mechanically, UL23 was discovered to impair the signal transducer and activator of transcription 1 (STAT1) phosphorylation, although it was not found to affect phosphorylation and expression of STAT2, Janus activated kinase 1, or tyrosine kinase 2, which are associated with IFN-I signal transduction pathway. Additionally, a significantly reduced nuclear expression of STAT1 but not of IFN regulatory factor 9 or STAT2 was observed. Findings of this study indicate that HCMV UL23 is a viral antagonist that acts against the cellular innate immunity and reveal a possible novel effect of UL23 on IFN-I signaling.

## Introduction

Human cytomegalovirus (HCMV), the human beta-herpesvirus, can induce severe syndromes among immunocompromised adult populations and newborns, such as HIV-infected populations and organ transplant recipients (Sweet, 1999; Hommes et al., 2004; Steininger, 2007; Cheeran et al., 2009). The HCMV infection rate is between 60 and 70% across developed countries and more than 90% across developing countries (Fulop et al., 2013). However, no approved HCMV vaccine is available to date, and the drugs used to treat HCMV infections are lowly bioavailable and may induce resistance (Biron, 2006). Consequently, the development of new antiviral management strategies to treat HCMV-induced infectious diseases is urgently required. HCMV represents the characteristic dsDNA virus encoding more than 200 proteins; these proteins can also regulate immune defenses in the host and exert an important part in viral pathogenic mechanism (Jarvis and Nelson, 2002; Goodrum et al., 2012; Noriega et al., 2012).

Congenital immunity represents the first line of defense in the host to resist many human viruses including HCMV (Stern-Ginossar et al., 2012; Tandon and Mocarski, 2012; Goodwin et al., 2018). Type I interferons (IFN-I), like IFN-β, account for the critical cytokines associated with immune surveillance that confer congenital immunity to resist viral infection (Platanias, 2005; Schoggins, 2019). IFN-I signal transduction pathways were identified as the critical factors that limit HCMV infection and replication (Dell’Oste et al., 2020). IFNs can bind to corresponding cell receptors and thus can activate the signal transduction pathways in cells. IFN-I can bind to IFNAR1/IFNAR2 (the subunits of IFN receptors) on the cell surface; in addition, tyrosine kinase 2 (Tyk2) and Janus activated kinase 1 (Jak1) can bind to receptor cytoplasmic tails (Pestka, 1997). Activation of JAKs related to the IFN-I receptor results in signal transducer and activator of transcription 1 (STAT1)/STAT2 tyrosine phosphorylation. Moreover, STAT1 and STAT2 can form the heterotrimeric transcription factor (TF) by binding to IFN regulatory factor 9 (IRF9), which has been called the IFN-stimulated gene factor 3 (ISGF3) complex (Platanias, 2005; Bonjardim et al., 2009). ISGF3 is then subjected to nuclear translocation, followed by binding to the IFN-stimulated response elements (ISREs) within DNA for driving diverse interferon-stimulated genes (ISGs) transcription, most of which can exert antiviral effects (Schneider et al., 2014; Platanitis et al., 2019).

Human cytomegalovirus acquires diverse proteins for antagonizing its antiviral activity, thus precluding IFN-I production or evading downstream IFN-I response during coevolution with the host (Browne and Shenk, 2003; Abate et al., 2004; Cristea et al., 2010; Li et al., 2013; Kim and Ahn, 2015; Fu et al., 2017, 2019; Kim et al., 2017; Biolatti et al., 2018; Choi et al., 2018; Huang et al., 2018; Park et al., 2019). For example, HCMV pp65 protein, encoded by *UL83* gene, was reported to reduce the activities of nuclear factor-κB (NF-κB), phosphorylation and re-localization of IRF3, and enzymatic activity of cyclic guanosine monophosphate-adenosine monophosphate (cGAS, a DNA sensor), leading to a reduced IFN-I gene expression (Browne and Shenk, 2003; Abate et al., 2004; Biolatti et al., 2018). We previously reported that the HCMV tegument protein, UL23, blocks antiviral type II interferons (IFN-II) responses through binding to *N*-Myc interactor protein (Nmi) in the human body (Feng et al., 2018). However, whether HCMV UL23 also targets the IFN-I response remains unclear. Furthermore, the mechanisms underlying UL23-mediated innate immune escape associated with IFN-I remain unclear.

The present study focused on exploring the UL23 effect on modulating IFN-I responses during HCMV infection. Herein, we provide evidence to confirm that HCMV UL23 antagonizes the IFN-β-induced antiviral action. We identified a key role of UL23 in conferring viral resistance to type I IFN during HCMV infection and in repressing the IFN-β-induced ISG expression. Notably, UL23 did not suppress the tyrosine phosphorylation in STAT2, JAK1, and TYK2 but suppressed the activation of STAT1 after treatment with IFN. In addition, UL23 selectively prevented IFN-β-induced nuclear translocation of STAT1 protein. Therefore, UL23 antagonizes IFN-β-stimulated signal transduction to substantially attenuate STAT1 protein phosphorylation and ISGF3 complex formation upon HCMV infection. Findings of this study suggest a novel role of UL23 in suppressing the IFN-I response, which also suggests a distinct mechanism through which HCMV evades the antiviral innate immunity of type I IFN.

## Materials and Methods

### Cells, Viruses, Reagents, and Antibodies

We obtained human foreskin fibroblasts (HFFs) from Lonza Inc. (Basel, Switzerland), whereas human glioblastoma HEK 293T and U251 cells were provided by the American Type Culture Collection. We cultivated the cells within Dulbecco’s modified Eagle’s medium (DMEM) (Invitrogen, Carlsbad, CA, United States) containing 10% fetal bovine serum (FBS) and 1% penicillin/streptomycin under 5% CO_2_ and 37°C temperature. HCMV (Towne, ΔUL23) was grown in human cells, according to previous description (Feng et al., 2018).

Recombinant human IFN-β (AF-300-02B-100) was purchased from PeproTech (Cranbury, NJ, United States). Rabbit anti-phosphor-STAT1 (9167), rabbit anti-STAT1 (14994), rabbit anti-phosphor-STAT2 (4441), rabbit anti-STAT2 (72604), mouse anti-HA (3724), and mouse anti-Flag (14793) were provided by Cell Signaling Technology, Inc. (Boston, MA, United States). Mouse antitubulin (66031-1-Ig), anti-STAT2 (16674-1-AP), anti-IRF9 (14167-1-AP), and rabbit anti-Jak1 (66466-1-Ig) were provided by (Manchester, United Kingdom) Rabbit anti-Tyk2 (A2128) was purchased from (Wuhan, China), rabbit anti-phosphor-Tyk2 (ab138394) was purchased from Abcam, Inc. (Cambridge, United Kingdom), and rabbit anti-phosphor-Jak1 (AF5857) was purchased from (Shanghai, China). Mouse anti-UL23 HCMV monoclonal antibodies and rabbit anti-UL23 HCMV polyclonal antibodies utilized in the present study are consistent with those described previously (Chai et al., 2019; Li et al., 2020).

### Lentiviral Transduction and Cell Line Generation

psPAX2 or pMD2.G plasmid was co-transfected in 293T cells with the UL23 retrovirus or control plasmid. After 24 h, we replaced the original medium with freshly prepared medium. After an additional 24 h, the supernatant was harvested and added to U251 cells with 8 μg/ml polybrene, followed by 24 h of incubation. Subsequently, 1 μg/ml puromycin was used to select infected cells 2 weeks prior to experiments.

### Viral Infection and Growth Analysis

Human foreskin fibroblast cells were used to infect virus stocks [multiplicity of infection (MOI) = 0.01]. The cells were further incubated until all cells displayed cytopathic effects. After removing debris and cells, the sample was preserved at –80°C until use. To quantify viral growth, we collected the medium and cells 5 days after infection, followed by preparation of virus stocks that were then gradually diluted before infection of HFF cells (1 × 10^5^). After agar overlay, we observed and calculated the formation of plaques approximately 14 days after infection. Thereafter, to determine IFN-β susceptibility, viral growth was analyzed by 2 h incubation with 100 U/ml human recombinant IFN-β prior to infection. Results from three separate experiments were averaged to obtain the final value.

### Luciferase Activity Assay

This study adopted the Dual-Luciferase Reporter (DLR) Assay system (Promega, Madison, WI, United States) for detecting luciferase activities in line with specific protocols. HEK293T cells reaching 70–80% confluence were grown in the 12-well plates overnight and then transfected with the pRL-TK *Renilla* luciferase expression plasmid, reporter plasmid ISRE luciferase, and specific expression plasmids. At 46 h post-transfection, 100 U/ml recombinant human IFN-β was used to treat cells for additional 2 h (or not). Cell lysates were used for DLR detection and Western blot analysis. The relative activity of firefly luciferase obtained from the results was normalized to that of *Renilla* luciferase.

### RNA Isolation and Quantitative Real-Time PCR

We utilized TRIZOL reagent (Invitrogen) for extracting total cellular RNA following specific protocols. Thereafter, 0.5–1 μg extracted RNA was prepared into complementary DNA (cDNA) with the cDNA synthesis kit (TaKaRa, Shanghai, China) through reverse transcription. The SYBR Green Supermix (Applied Biosystems, Inc., Foster City, CA, United States) was employed for the quantitative real-time PCR (qRT-PCR) assay conducted with specific primers. Oligonucleotide primers utilized were shown below: for *CXCL10*, forward 5′-CGCTGTACCTGCATCAGCAT-3′, reverse 5′-GCAATGATCTCAACACGTGGAC-3′; for *MX1*, forward 5′-ACATCCAGAGGCAGGAGACAATC-3′, reverse 5′-TCCACCAGATCAGGCTTCGTCAA-3′; for *OAS1*, forward 5′-CCAAGCTCAAGAGCCTCATC-3′, reverse 5′-TGGGCTGT GTTGAAATGTGT-3′; and for *GAPDH*, forward 5′-GAAG GTGAAGGTCGGAGTC-3′, reverse 5′-AAGATGGTGATGG GATTTC-3′. The relative target gene level was calculated based on the *GAPDH* messenger RNA (mRNA) level. Data from three individual experiments were obtained for the analysis and are displayed as mean ± SD.

### Immunoblotting and Coimmunoprecipitation Assays

Cells were collected using radioimmunoprecipitation assay (RIPA) lysis buffer (Sigma Aldrich, St. Louis, MO, United States) containing the protease inhibitor cocktail (Roche, Basel, Switzerland). Thereafter, sodium dodecyl sulfate–polyacrylamide gel electrophoresis (SDS-PAGE) was performed to separate aliquots of proteins, followed by transfer of the proteins on polyvinylidene fluoride (PVDF) membranes (Millipore, Bedford, MA, United States). Afterward, 5% skimmed milk contained in *Tris*-buffered saline with Tween 20 (TBST) solution was used to block membranes, and specific primary antibodies were used to incubate membranes overnight under 4°C. After washing the membranes thrice with TBST, membranes were further incubated with horseradish peroxidase (HRP)-labeled secondary antibodies for another 2 h. Subsequently, the Western chemiluminescent substrate kit (Thermo Fisher, Waltham, MA, United States) was adopted to visualize bands, and the Gel Documentation Station (BioRad, Hercules, CA, United States) was utilized for quantification.

We adopted protein A/G immunoprecipitation kits (Cell Signaling Technology Inc.) for coimmunoprecipitation (Co-IP) assays. Cells were collected using protease inhibitor cocktail-containing RIPA lysis buffer; using 10% lysates as the input reference, we added primary antibodies directly into cell lysis buffer with protein A/G and purified according to specific instructions. Thereafter, SDS-PAGE was performed to separate precipitated proteins, followed by transfer of the proteins on the membranes. Then, primary antibodies were used to incubate the membranes. Western chemiluminescent substrate kit was adopted for staining, whereas the Gel Documentation Station was adopted for quantification of the proteins.

### Immunofluorescence Assays

For immunofluorescent staining, cells were fixed with 4% paraformaldehyde, followed by 0.2% Triton X-100 permeabilization and incubation with specific primary and secondary antibodies. Afterward, 4′,6-diamidino-2-phenylindole (DAPI) was adopted to stain cell nuclei. A Nikon Eclipse TE2000-S microscope (Nikon, Tokyo, Japan) was employed to observe the cells. Meanwhile, a Leica TCS SP8 microscope (Leica Microsystems, Wetzlar, Germany) was used to collect confocal images from individual channels. Finally, Leica Microsystems was utilized to merge all digital images.

### Statistical Analysis

Each assay was performed thrice, and comparable outcomes were obtained. Unpaired Student’s *t*-test (two-sided) was adopted to assess significant differences between the two groups. A difference of *p* < 0.05 suggested statistical significance. Graphs present the mean ± SD value, with *n* = 3. For unpaired *t*-test, ^∗^*p* < 0.05; ^∗∗^*p* < 0.01; and ^∗∗∗^*p* < 0.001.

## Results

### HCMV UL23 Confers Resistance to IFN-I in the Case of Viral Infection

**FIGURE 1:**
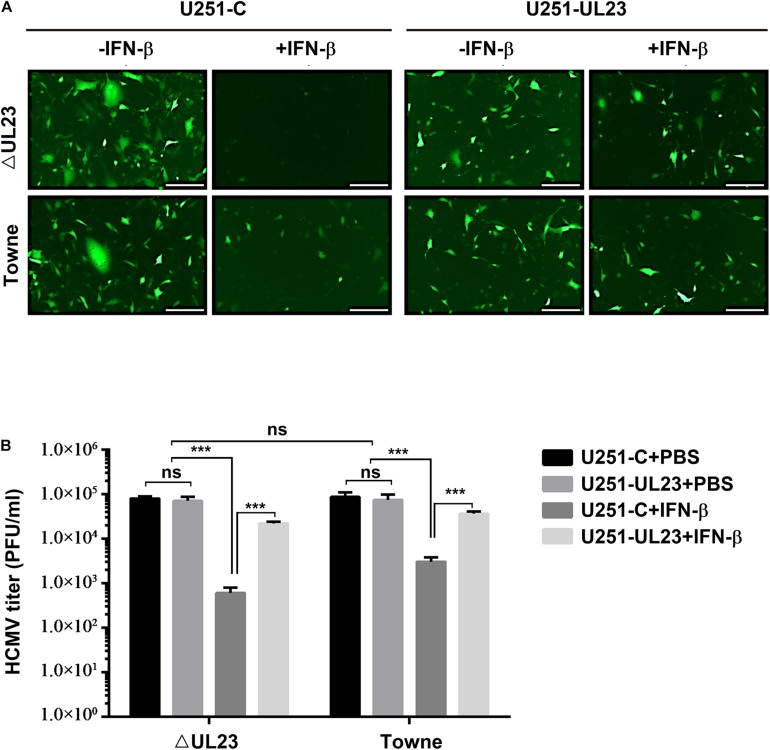
Human cytomegalovirus (HCMV) UL23 confers resistance to type I IFN upon viral infection. **(A)** UL23 stably expressed U251 cells (U251-UL23) and control cells (U251-C) were treated with or without 100 U/ml IFN-β for a period of 2 h and then infected with HCMV or HCMV–ΔUL23 (MOI = 1) for 72 h before fluorescence microscopy. Scale bars, 200 μm. **(B)** After infection for 5 days, we harvested the total infected cell cultures, followed by determination of viral titers. Each assay was conducted thrice. Data are shown as mean ± SD. *p < 0.05, **p < 0.01, ***p < 0.001 (unpaired, two-tailed Student’s *t*-test). Data are representative of three independent experiments with similar results.

It has been previously suggested that HCMV infection and replication are suppressed within cells treated with IFN-β (Sainz et al., 2005; Paulus et al., 2006). To elucidate the HCMV UL23 effect on the IFN-β anti-HCMV action, we analyzed the exogenous IFN-β susceptibility of UL23-deficient HCMV (ΔUL23) and wild-type HCMV (Towne). First, U251-Flag-UL23 or U251-C cells were transfected with pCDNA-Flag-UL23 and pCDNA with/without IFN-β. Afterward, HCMV ΔUL23 or HCMV Towne (MOI = 1) was utilized to infect cells for 3 or 6 days. Infected U251-UL23 and U251-C cells were then examined under a fluorescence microscope. In IFN-β-treated U251-C cells, HCMV ΔUL23 virus generation was found to be suppressed, whereas Towne virus production was found to be attenuated but not fully blocked by IFN-β (Figure 1A, left). However, in IFN-β-mediated U251-UL23 cells, no difference was observed between HCMV ΔUL23 replication and HCMV Towne replication (Figure 1A, right). These results indicated that the IFN-β susceptibility of HCMV ΔUL23 virus increased relative to HCMV Towne virus.

The UL23 protein effect on viral replication in response to IFN-β action was further evaluated in U251–UL23 and U251-C cells. Notably, without IFN-β, the viral titters of HCMV ΔUL23 were not found to differ between the above two cells, whereas with IFN-β, the viral titters of HCMV ΔUL23 were found to be significantly increased (approximately 100-fold) within U251–UL23 cells relative to U251-C cells (Figure 1B, left), suggesting that UL23 exerts a vital part in suppressing the antiviral function of IFN-β. Likewise, without IFN-β, no difference was noted in the viral titters of HCMV Towne between U251-C cells and U251–UL23 cells, whereas after IFN-β treatment, Towne virus had mildly increased viral titters (approximately 10-fold increased titers) in U251–UL23 cells relative to U251-C cells (Figure 1B, right), which verified the involvement of UL23 in the repression of IFN-β antiviral function. Our results also showed that before IFN-β treatment, viral titters in both U251-C cells and U251–UL23 cells were not significantly different between HCMV Towne and HCMV ΔUL23 virus (Figure 1B, right). This result conformed to prior findings, suggesting an essential role of UL23 in viral lytic infections (Dunn et al., 2003). Moreover, the results indicated that in U251–UL23 cells, both HCMV ΔUL23 and HCMV Towne were insensitive to IFN-β treatment and exhibited high viral titters with IFN-β (Figure 1B), suggesting that ectopic UL23 expression represses IFN-β action upon HCMV infection. Collectively, these results indicated that HCMV UL23 protein exerts an important part in suppressing IFN-β response while increasing viral resistance to IFN-β upon viral infection.

### UL23 Represses Type I IFN-Induced Transcription of ISGs During HCMV Infection

**FIGURE 2:**
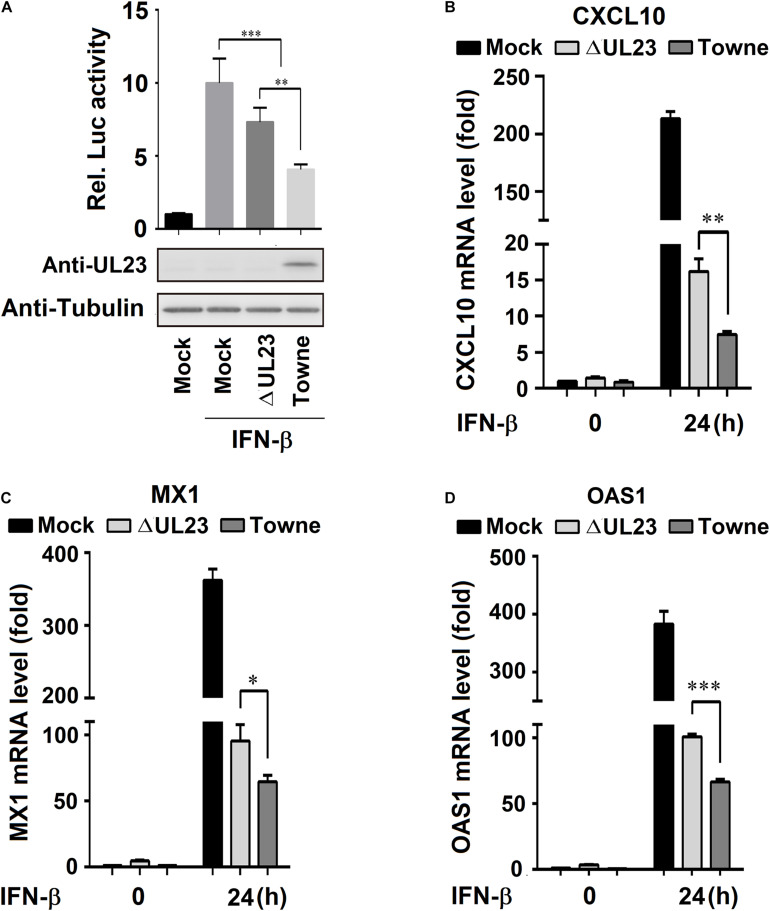
UL23 represses type I IFN-induced transcription of IFN-stimulated genes (ISGs) during HCMV infection. **(A)** U251 cells were cotransfected with ISRE-Luc reporter and pRL-TK reporter. At 24 h post-transfection, cells were infected with mock or with HCMV or HCMV–ΔUL23 (MOI = 3) for 24 h before luciferase assays. UL23 expression was measured through the Western blot assay, with Tubulin as a loading reference. **(B–D)** HCMV-ΔUL23 or WT HCMV (MOI = 3) was used to infect U251 cells for 24 h, followed by 100 U/ml IFN-β treatment for 24 h. The **(B)** *CXCL10*, **(C)** *MX1*, **(D)** and *OAS1* mRNA expression was measured through qRT-PCR, with *GAPDH* as the reference. Each assay was conducted thrice. Data are shown as mean ± SD. *p < 0.05, **p < 0.01, ***p < 0.001 (unpaired, two-tailed Student’s *t*-test). Data are representative of three independent experiments with similar results.

For the activation of IFN-I-mediated transcription of ISGs, the nuclear translocation of ISGF3, the tripartite TF comprising IRF9, STAT1, and STAT2 are necessary to recognize ISRE promoters of these ISGs (Sadler and Williams, 2008; Gonzalez-Navajas et al., 2012). The present study aimed to determine the UL23 role in modulating IFN-β-mediated interaction between ISGF3 and sequence-specific ISRE promoter elements in the process of HMCV infection. pRL-TK and pGL3-promoter-ISRE plasmids were transfected into U251 cells, followed by IFN-β treatment and HCMV infection. IFN-β significantly promoted the activities of ISRE within mock-treated U251 cells, whereas IFN-β-induced ISRE activities were reduced in HCMV ΔUL23-infected U251 cells and significantly inhibited in HCMV Towne-infected U251 cells (Figure 2A, top). We noted a lack of expression of UL23 protein within U251 cells infected with HCMV ΔUL23 or mock; however, UL23 protein was found to express only in HCMV Towne-infected U251 cells (Figure 2A, bottom). These findings indicated that UL23 protein suppresses the modulation of IFN-β-mediated ISRE activity. We further explored whether UL23 protein represses antiviral activity of IFN-I through suppressing the ISGs activation upon HCMV infection. Results indicated that the IFN-β-induced ISGs mRNA expression (*CXCL10*, *MX1*, and *OAS1*) was significantly stimulated by IFN-β in mock-infected U251 cells, although the levels of IFN-β-induced ISGs were reduced in HCMV ΔUL23-infected U251 cells and significantly inhibited in HCMV Towne-infected U251 cells (Figures 2B–D). Collectively, these results indicated the role of UL23 protein in reducing ISG expression mediated by IFN-β upon HCMV infection.

### Ectopic Expression of UL23 Represses IFN-β-Induced Transcription of ISGs

**FIGURE 3:**
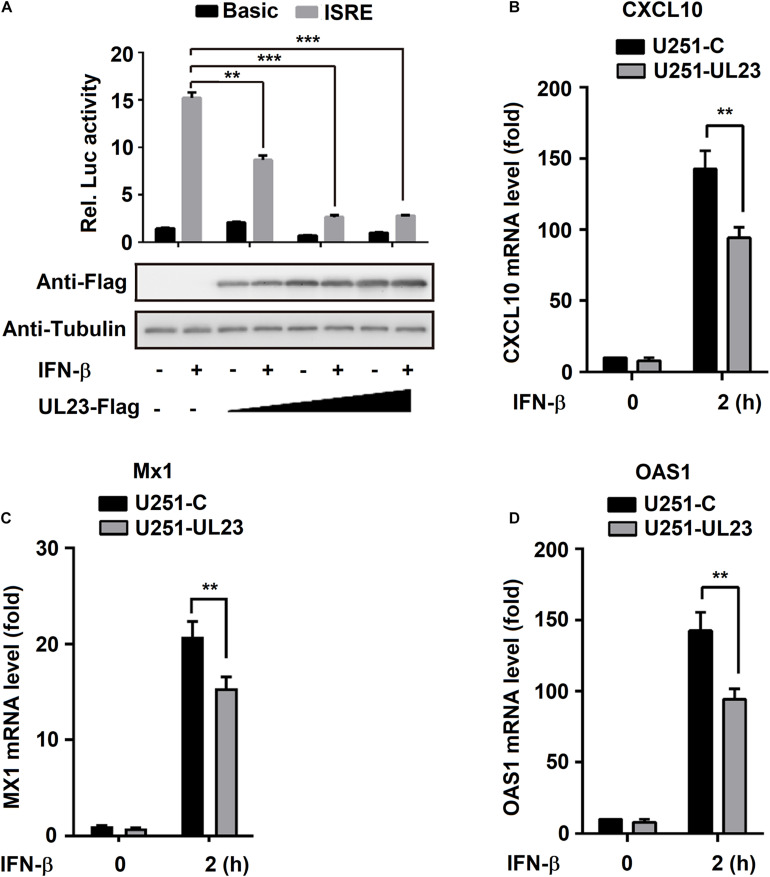
Inhibition of IFN-β induces transcription by ectopic expression of UL23. **(A)** pRL-TK reporter or ISRE-Luc reporter was cotransfected into HEK293T cells with UL23 expression plasmid or empty vector. After transfection for 46 h, IFN-β (100 U/ml) was used to treat cells for 2 h or not treated, and then, luciferase activity was measured luminometrically by using the Dual-Luciferase reporter assay as relative light units. UL23 expression was measured through WB assay, with *Tubulin* as the loading reference. **(B–D)** qRT-PCR analysis of **(B)** *CXCL10*, **(C)** *MX1*, and **(D)** *OAS1* expression in U251-C and U251-UL23 cells stimulated with 100 U/ml IFN-β for the indicated time points. Data are shown as mean ± SD. *p < 0.05, **p < 0.01, ***p < 0.001 (unpaired, two-tailed Student’s *t*-test). Data are representative of three independent experiments with similar results.

For better determining HCMV UL23 protein effect on modulating IFN-β-induced ISGs, this study overexpressed UL23 in the cells. Initially, pGL3-promoter-ISRE was cotransfected into HEK293T cells with pRL-TK, and this step was followed by IFN-β treatment and pUL23-Flag plasmid transfection at different concentrations as indicated. Consequently, the activity of ISRE was induced by IFN-β as expected; however, IFN-β-induced ISRE activity was attenuated by UL23 within HEK293T cells dose dependently (Figure 3A). Besides, U251-UL23 and U251-C cells were either exposed to IFN-β or not. Our results indicated that although the mRNA expressions of *CXCL10*, *OAS1*, and *MX1* are induced by IFN-β, IFN-β-induced *CXCL10*, *MX1*, and *OAS1* mRNA expressions were not affected within U251-C cells but repressed within U251-UL23 cells (Figures 3B–D). These results implied that the ectopic expression of UL23 represses the IFN-β-mediated ISG mRNA expression and ISRE promoter activity.

### UL23 Attenuates IFN-β-Mediated Phosphorylation of STAT1

**FIGURE 4:**
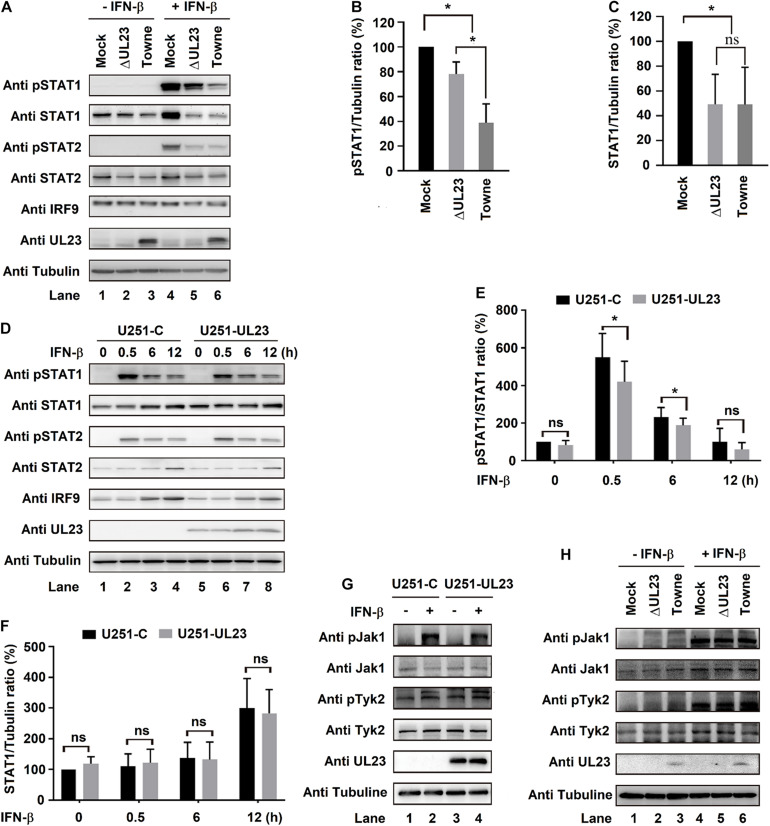
UL23 attenuates IFN-β-mediated phosphorylation of the signal transducer and activator of transcription 1 (STAT1). **(A–C)** U251 cells infected with HCMV Towne or HCMV–ΔUL23 (MOI = 3) for 72 h. The cells were then treated with IFN-β (100 U/ml) for 0.5 h before **(A)** immunoblot analysis, **(B)** phosphorylation detection, **(C)** and protein determination. **(D–F)** U251–UL23 cells and U251-C cells were exposed to 100 U/ml IFN-β for appropriate time periods prior to the **(D)** immunoblot assay, **(E)** phosphorylation detection, and **(F)** protein determination. **(G)** IFN-β (100 U/ml) was used to treat U251–UL23 and U251-C cells for a period of 0.5 h prior to the immunoblot assay. **(H)** HCMV–ΔUL23 or HCMV (MOI = 3) was transfected into U251 cells for a period of 72 h, followed by 0.5 h of 100 U/ml IFN-β treatment prior to the immunoblot assay. Data are shown as mean ± SD. *p < 0.05, **p < 0.01, ***p < 0.001 (unpaired, two-tailed Student’s *t*-test). Data are representative of three independent experiments with similar results.

Upon IFN signaling stimulation, STAT phosphorylation occurs at the C-terminal of tyrosine residues (Y701 and Y690, respectively, in STAT1 and STAT2), and then, STAT1 and STAT2 bind to IRF9 to form ISGF3 complex (Borden et al., 2007; Cheon et al., 2013). Phosphorylation levels of IRF9, STAT1, and STAT2 were shown to decrease after HCMV infection (Miller et al., 1999). In this study, we determined whether UL23 exerts a vital part in ISGF3 components phosphorylation and expression (STAT1, STAT2, and IRF9). HCMV Towne or HCMV ΔUL23 virus was used to infect U251 cells after IFN-β treatment. In mock-infected cells, phosphorylated STAT1 protein (pSTAT1), phosphorylated STAT2 protein (pSTAT2), steady-state STAT1 protein (STAT1), steady-state STAT2 protein (STAT2), and steady-state IRF9 protein (IRF9) proteins were induced by IFN-β (Figure 4A, lane 4 vs. 1). We noted that IFN-β-induced levels of pSTAT1 and pSTAT2 proteins were reduced in HCMV Towne-infected cells (Figures 4A,B, lane 6 vs. 4) but significantly repressed in HCMV ΔUL23-infected cells (Figure 4A,B, lane 5 vs. 4), although IFN-β-induced levels of STAT1, pSTAT2, and IRF9 proteins were attenuated to similar degrees in both HCMV Towne- and HCMV ΔUL23-infected cells (Figures 4A,C, lanes 5 and 6). These results indicated that HCMV UL23 protein participates in the repression of IFN-β-induced STAT1 phosphorylation, yet it was not found to affect the IFN-β-regulated STAT1, STAT2, and IRF9 expression.

Moreover, U251-UL23 and U251-C cells were exposed to IFN-β or not at diverse time points. Consistently, as a result, IFN-β-induced phosphorylated STAT1 protein (pSTAT1) level significantly decreased within U251-UL23 cells compared with that within U251-C cells (Figures 4D,E, lanes 6, 7 vs. 2, 3), whereas the levels of IFN-β-induced steady-state STAT1 protein (STAT1), steady-state STAT2 protein (STAT2), and steady-state IRF9 protein (IRF9) were relatively unchanged in both U251-C cells and U251-UL23 cells (Figures 4D,F, lanes 6, 7 vs. 2, 3). These data confirmed that HCMV UL23 protein participates in attenuating the IFN-β-mediated STAT1 phosphorylation, yet it was not found to affect the IFN-β-mediated expressions of IRF9, STAT1, and STAT2.

We further investigated the role of UL23 in regulation of the phosphorylation cascade components (Jak1 and Tyk2) upon IFN-I signaling stimulation. U251–UL23 and U251-C cells were either subjected to IFN-β treatment or left untreated. As anticipated, the levels of pJak1 and pTyk2 but not of Jak1 and Tyk2 were robustly promoted through IFN-β within both U251 cells (Figure 4G, lanes 2 vs. 1) and U251–U23 cells (Figure 4G, lanes 4 vs. 3). Additionally, the levels of pJak1, pTyk2, Jak1, and Tyk2 remained unchanged in both U251 cells and U251–U23 cells (Figure 4G, lanes 2 and 4 vs. 1 and 3). In addition, HCMV Towne and HCMV ΔUL23 were used to infect U251 cells, followed by treatment with or without IFN-β. Levels of pJak1/pTyk2 were markedly stimulated by IFN-β, as expected (Figure 4H, lanes 4–6 vs. 1–3). Additionally, the levels of IFN-β-stimulated pJak1 and pTyk2 remained unchanged in both HCMV ΔUL23- and HCMV Towne-infected U251 cells (Figure 4H, lanes 6 vs. 5). These data indicated that UL23 causes no difference in IFN-β-induced Jak1/Tyk2 phosphorylation. Collectively, these results indicated a role of UL23 in specifically attenuating the IFN-β-regulated STAT1 phosphorylation.

### UL23 Destroys STAT1 Nuclear Localization Upon IFN-I Treatment

**FIGURE 5:**
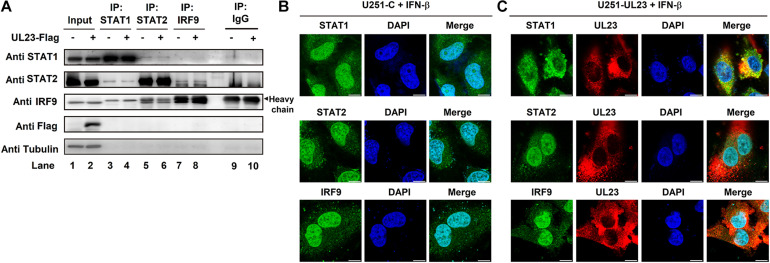
UL23 disrupts the nuclear localization of STAT1 in response to type I IFN. **(A)** Empty vector or Flag-UL23 expression plasmid was transfected into HEK-293T cells. After transfection for 46 h, the lysed cells were subjected to immunoprecipitation by using anti-IRF9, anti-STAT1, and anti-STAT2 antibodies, separately, and then to immunoblotting by using specific antibodies; 100 U/ml IFN-β was then used to treat **(B)** U251-C cells and **(C)** U251–UL23 cells for a period of 0.5 h. After fixation, antibodies were used to stain the cells, as indicated. The images of UL23 (red), STAT1 (green), STAT2 (green), and IRF9 (green) were merged with DAPI-stained nuclei (blue) to generate composite images. Scale bars, 10 μm.

To maintain a high expression of ISGs, ISGF3 must complete nuclear translocation and then combine with ISRE or GAS elements within corresponding promoters following IFN exposure (Platanias, 2005; Cheon et al., 2011). We further speculated that UL23 represses ISGF3-induced ISRE promoter activity by directly blocking the association among IRF9, STAT1, and STAT2. To evaluate the aforementioned hypothesis, we conducted co-IP assays to examine the relationship of UL23 with ISGF3 components (STAT1, STAT2, and IRF9). HEK293T cells were transfected into empty vector or Flag-UL23 expression plasmid. First, we utilized anti-IRF9, anti-STAT1, and anti-STAT2 antibodies for immunoprecipitate protein lysates collected in transfected cells, followed by immunoblotting performed using antibodies, as indicated. Results indicated the coimmunoprecipitation of STAT1–STAT2 and STAT2–IRF9 complexes in cell extracts, as reported previously (Platanitis et al., 2019); however, no interaction between UL23 and ISGF3 subunits was observed in the co-IP assay (Figure 5A).

Furthermore, we examined whether UL23 influences the nuclear/cytoplasmic distributions of IRF9, STAT1, and STAT2 within IFN-β-treated U251–UL23 and U251 cells depending on the signaling. Our result indicated that STAT1, STAT2, and IRF9 proteins are translocated into the nucleus of U251-C cells upon IFN treatment (Figure 5B), conforming to the results of previous studies (Platanias, 2005; Wiesauer et al., 2015; Wang et al., 2019). Consistent with previous results (Adair et al., 2002; Feng et al., 2018), this study indicated that UL23 localizes in the cytoplasm of cells treated with IFN-β (Figure 5C). Importantly, STAT1 mainly exhibited cytoplasmic localization in U251–UL23 cells; however, cytoplasmic/nuclear distributions of STAT2 and IRF9 were not found to be affected by UL23 (Figure 5C). The above results suggested that UL23 exerts a vital part in repressing IFN-β-induced STAT1 translocation but has no role in IFN-β-induced cellular distributions of STAT2 and IRF9. Collectively, the results indicated that UL23 specifically prevents IFN-β-mediated STAT1 nuclear accumulation.

## Discussion

Interferon response accounts for the first line of defense to resist HCMV infection by producing hundreds of antiviral ISGs that restrict the viral replication (Platanias, 2005; Sadler and Williams, 2008; Gonzalez-Navajas et al., 2012; Schoggins, 2019). HCMV acquires various proteins for antagonizing antiviral activities, thus precluding IFN-I production or evading the downstream IFN-I response. Nonetheless, except IE, none of the proteins encoded by herpesviruses can suppress IFN-I immune responses to achieve immune evasion. Yet, our knowledge of the mechanism through which HCMV mediates host IFN-I response during infection remains exceedingly sparse. This work recognized HCMV UL23 protein as the crucial factor for negatively regulating IFN-I immune responses. Our results revealed that several crucial signal transduction steps, such as the activation and phosphorylation of STAT1, nuclear localization of STAT1, and activity of the ISGF3-induced ISRE, are disrupted by UL23 during HCMV infection, which leads to the inhibition of transcription of downstream ISGs (*CXCL10*, *MX1*, and *OAS1*) as well as an increase in viral resistance to IFN-β.

Studies have shown that the exposure of HFFs to either IFN-β or IFN-γ suppresses plaque formation in HCMV by approximately 30–40 times, whereas the treatment combining IFN-β with IFN-γ suppresses plaque formation by 662 times, indicating that IFN-β and IFN-γ act via distinct pathways (Sainz et al., 2005). Conforming to these results, in our study, viral titters were found to decrease by 20 times after IFN-β treatment in HCMV-infected U251 cells compared with those in untreated cells. Through signaling on certain cell surfaces, IFN finally mediates diverse ISG expressions, some of which have been found to exert antiviral effects. Recently, some herpesviral antagonists were demonstrated to target ISGs (Gonzalez-Perez et al., 2020). *CXCL10*, one of the proinflammatory cytokines, participates in various processes such as apoptosis, chemotaxis, and differentiation (Liu et al., 2011). *CXCL10* was suggested as a vital factor for resisting herpes simplex virus infection in the host (Lokensgard et al., 2001). *MX1* expression in IFN-treated cells was identified to suppress several virus replications through interfering with the traffic or synthesis of the viral machinery (Haller and Kochs, 2011). OAS proteins are the well-studied ISG effectors that can directly degrade viral RNAs or viral genomes (Kristiansen et al., 2011). Previous investigations have demonstrated that the viral infection of fibroblasts induces the expression of ISGs, which may be due to the activation of antiviral immunity and secretion of IFN-I, although these processes occur during early viral infection (Fu et al., 2017, 2019; Zou et al., 2020). As for the virus replicates, it inhibits ISGs to build an environment, conducive to its replication and proliferation, through various evolutionary mechanisms. Inhibition of *CXCL10*, *MX1*, and *OAS1* by UL23 at late times after HCMV infection was observed in this study, so UL23 protects HCMV against the immunomodulatory and antiviral responses of IFN-I. Investigating UL23 effect on fibroblasts, which have been frequently utilized to investigate HCMV infection, may be useful (Mocarski et al., 2007). However, the low transfection efficiency of fibroblasts presents technical difficulties for conducting resemble experiments among such cells. In addition, the constitutive high expression of UL23 through numerous passages within fibroblasts is not feasible since the fibroblast cells are primary cells. On the contrary, U251 cells of neuronal origin allow for HCMV infection and thus are extensively adopted for studying HCMV infection (Trang et al., 2000; Mocarski et al., 2007; Pei et al., 2012).

Clearly, HCMV has evolved different efficient immune escape mechanisms to counteract the host IFN response, as described previously (Dell’Oste et al., 2020). Studies have also indicated that HCMV IE1 protein inhibits the transcription of ISGs by sequestering its binding of promyelocytic leukemia (PML) and STAT2, thus decreasing IFN responses while increasing IFN-β tolerance in the virus (Paulus et al., 2006; Kim and Ahn, 2015). UL23 does not directly interact with ISGF3 subunits or limit tyrosine phosphorylation of STAT2; however, it selectively inhibits STAT1 activation in response to IFN-β treatment. Perhaps UL23 inhibits STAT1 phosphorylation by blocking the expression and phosphorylation of Jak1 or Tyk2 because the phosphorylation of Jak1 or Tyk2 occurs before STAT activation. However, Jak1 or Tyk2 phosphorylation and expression were not affected by UL23, and one possible explanation is the indirect involvement of UL23 in the repression of JAK/STAT signaling pathway through the host factor possibly mediated by IFN-I. Future studies should adopt proteomic methods such as tandem mass tag labeling combined with mass spectrometry to comprehensively analyze UL23-modulated, IFN-I-triggered proteins of ΔUL23 mutant- or Towne virus-infected cells. The above results help to identify potential factors, including UL23-regulated cytokine signaling suppressors and protein tyrosine phosphatases.

To facilitate the nuclear translocation of STAT1 dimers, STAT1 should be phosphorylated and activated beforehand at the tyrosine 701 residue (Shuai et al., 1993, 1994; Wenta et al., 2008). As revealed by our findings, UL23 mainly suppresses STAT1 activation but does not directly reduce the protein level of STAT1. However, neither STAT2 nor IRF9 expression is affected by UL23 in IFN-β treated cells. According to the above findings, the ectopic UL23 expression hinders the nuclear import of STAT1 and promotes its cytoplasmic localization, although it exerts no remarkable effect on the retention of STAT2 and IRF9. STAT1 has been reported to localize primarily in the nuclei following IFN treatment (Platanias, 2005; Cheon et al., 2011). STAT1 phosphorylation exerts a vital part in nuclear localization; however, the nuclear transport mechanism remains unclear (Darnell, 1997; Levy and Darnell, 2002; Schneider et al., 2014).

**FIGURE 6:**
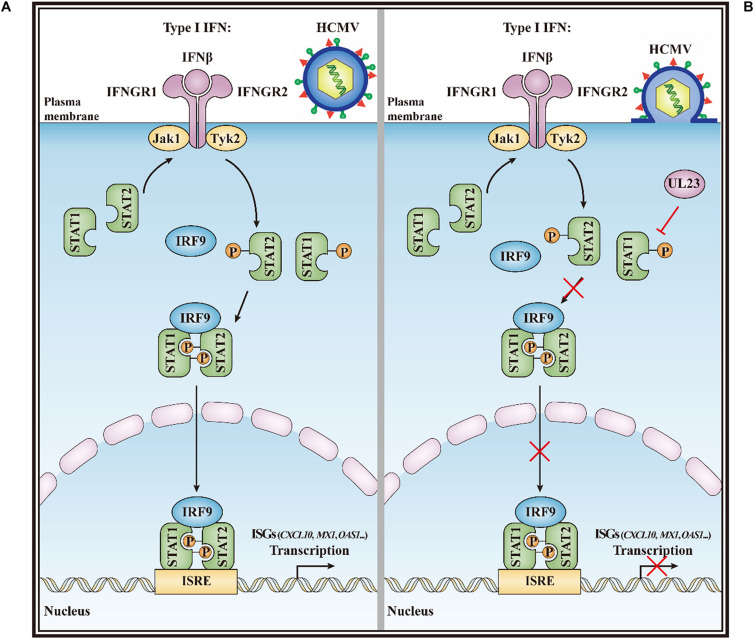
A model for the HCMV UL23-mediated suppression of type I interferon response. **(A)** IFN-I can trigger Tyk2 and Jak1 by binding to IFNAR1 and IFNAR2, separately, after recognizing the cognate receptor (IFNAR1/2). In turn, Tyk2 and Jak1 contribute to the tyrosine phosphorylation of receptor chains (Y–P), thus creating docking sites for the phosphorylation of STAT1 and STAT2. Thereafter, IRF9, STAT1, and STAT2 form the ISGF3 transcriptional regulator. And ISGF3 translocate to the nucleus for promoting transcription by the ISRE regulatory sequence in ISGs (such as *CXCL10*, *MX1*, and *OAS1*). **(B)** During HCMV infection, UL23 disrupts the STAT1 phosphorylation and nuclear localization of STAT1 in type I interferon signaling; inactivation of STAT1 by UL23 blocks phosphorylated ISGF3, reduces ISG transcription and ISRE promoter activation, prevents type I interferon-induced responses, and facilitates viral immune escape from type I interferon.

In summary, our study demonstrates that UL23 disrupts STAT1 phosphorylation and suppresses IFN-I response after HCMV infection. Thus, we propose a model by which UL23 attenuates STAT1 phosphorylation upon IFN-I stimulation upon HCMV infection (Figure 6). In this model, UL23 selectively blocks the phosphorylation of STAT1, leading to the repression of ISGF3 complex formation, reduction in STAT1 nuclear accumulation, and suppression of ISG expression. More investigations on the aforementioned aspects can help in elucidating the role of UL23 in interfering with IFN-I/IFN-II responses in the host, which can also facilitate HCMV replication and infection. These studies would help in elucidating the underlying mechanism of the action of UL23 as well as corresponding binding partners upon IFN-I exposure. Therefore, understanding the role of UL23 in type I IFN would enhance our understanding on the mechanism of how human viruses modulate type I IFN responses to evade intrinsic and innate defenses. The study also offers a candidate management strategy for preventing and treating viral infections in humans.

## Data Availability Statement

The original contributions presented in the study are included in the article/supplementary material, further inquiries can be directed to the corresponding authors.

## Author Contributions

LF, JW, and HL contributed to the study concept and design. LF, WL, XW, and XL carried out most of the experiments. LF and WL analyzed the data with help from XY and YR. LF and HL contributed to the drafting of the manuscript. JW and HL contributed to editing the manuscript. All authors contributed to the article and approved the submitted version.

## Conflict of Interest

The authors declare that the research was conducted in the absence of any commercial or financial relationships that could be construed as a potential conflict of interest.
